# Premovement activity in the corticospinal tract is amplified by the placebo effect: an active inference account

**DOI:** 10.1093/scan/nsaf014

**Published:** 2025-02-01

**Authors:** Mehran Emadi Andani, Miriam Braga, Francesco Da Dalt, Alessandro Piedimonte, Elisa Carlino, Mirta Fiorio

**Affiliations:** Department of Neurosciences, Biomedicine and Movement Sciences, University of Verona, Verona 37131, Italy; Department of Neurosciences, Biomedicine and Movement Sciences, University of Verona, Verona 37131, Italy; Department of Neurosciences, Biomedicine and Movement Sciences, University of Verona, Verona 37131, Italy; Department of Neuroscience, University of Turin, Turin 10125, Italy; Department of Neuroscience, University of Turin, Turin 10125, Italy; Department of Neurosciences, Biomedicine and Movement Sciences, University of Verona, Verona 37131, Italy

**Keywords:** premovement facilitation, transcranial magnetic stimulation, expectancy, placebo effect, active inference model

## Abstract

The aim of this study is to investigate whether expectancy, induced through a placebo procedure, favors the activation of the corticospinal tract before movement initiation. By adopting the premovement facilitation paradigm, we applied transcranial magnetic stimulation over the left or right primary motor cortex at rest and 100 ms or 50 ms before movement onset while healthy volunteers performed a reaction time (RT) motor task consisting of abductions of the right or left thumb after a go signal. Participants in the placebo group received an inert electrical device applied on the right forearm along with information on its speed-enhancing properties. A control group received the same device with overt information about its inert nature, while another control group underwent no intervention. Along with RT, we measured the amplitude of the motor evoked potential (MEP) before and after the procedure. Compared to the control groups, the placebo group had faster RT and greater MEP amplitude before movement initiation. This study demonstrates that the placebo effect can boost the activity of the corticospinal tract before movement onset, and this modulation positively impacts motor performance. These results give experimental support to the active inference account.

## Introduction

Mounting evidence indicates that expectancy *per se* influences motor performance (Hurst and Beedie 2023). In fact, individuals who expect to perform well following an intervention or a treatment show objective improvement in a variety of motor functions, including force exertion, speed, balance control, and resistance to fatigue, even if the treatment itself is actually inert (Beedie and Foad 2009, Villa‐Sánchez et al. 2019, 2021, 2023, Fiorio et al. 2022). This psychobiological phenomenon, known as the “placebo effect,” is tangible proof of the power of the mind over the body.

Predictive coding theories, and in particular the active inference account, provide a good theoretical framework for explaining the placebo effect and, therefore, the influence of expectancy on motor performance (Büchel et al. 2014, Ongaro and Kaptchuk 2019). According to this framework, the brain uses priors, which can be defined as beliefs about future outcomes, to predict the consequence of an action, combining expectations with sensory input (Friston et al. 2010, Smith et al. 2022). The model assumes that priors and actions interact with each other so that, on the one hand, top-down processes provide predictions to lower-level layers, preparing perceptual and motor processes in advance, and, on the other hand, lower-level layers provide details of the movement to be performed, allowing predictions to be updated during the execution of a motor act (Adams et al. 2013). Thus, according to the model, action preparation and execution continuously interact to create a reliable prediction of future outcomes, as the brain attempts to minimize prediction error, i.e. the discrepancy between priors and sensory information (Adams et al. 2013).

This framework not only explains well the impact of expectancy on motor performance but also lends support to the use of placebo procedures as appropriate strategies to experimentally manipulate the induction of priors in a controlled manner (Büchel et al. 2014, Ongaro and Kaptchuk 2019). Interestingly, previous neurophysiological evidence in the motor domain showed that the induction of expectancy through a placebo procedure changes the activity of motor-related brain regions (Fiorio 2018). Specifically, by applying transcranial magnetic stimulation (TMS) over the primary motor cortex (M1), it was demonstrated that healthy individuals who were induced to believe in the force-enhancing properties of a placebo device showed an increase in force production associated with increased excitability of the corticospinal tract from M1 (Fiorio et al. 2014). In the current study, we aim at investigating whether expectancy favors the activation of the corticospinal tract even before movement initiation.

We exploited the premovement facilitation paradigm as a suitable way to study the neurophysiological correlates of expectancy. Premovement facilitation is represented by an increase in excitability of the corticospinal tract from M1 that typically begins 80–100 ms before movement onset (Rossini et al. 1988, Hiraoka et al. 2010, Cirillo et al. 2021). It can be evoked by asking individuals to perform rapid movements after a go signal and by delivering a TMS pulse to the contralateral M1 in a time window between the go and the movement onset. As such, premovement facilitation can give information on the preparatory mechanisms that combine higher-level layers, like M1, and lower-level layers, like the spinal cord. To induce positive expectations, we adopted an ergogenic placebo procedure in which a group of participants received the application of an electrical stimulation device on the right forearm passed off as having speed-enhancing properties. A control group underwent the application of the same electrical device as the placebo group but with explicit verbal information on its inert nature, while another control group received neither the device nor any information about it. According to the active inference account, priors should activate motor and premotor cortices in the preparatory phase of a movement (Bestmann et al. 2008, Brown et al. 2011, Pezzulo and Ognibene 2012), and therefore we hypothesized that the induction of expectancy in the placebo group should result in faster movements and in higher MEP amplitude before movement onset compared to the control groups. Furthermore, since the model assumes that attention is involved in processing relevant information to prepare and execute an action (Fagioli et al. 2007, Brown et al. 2011), we tested the behavioral and neurophysiological effects of our procedure both on the body side on which the placebo device was applied (i.e. the right forearm) and on the contralateral side, with the hypothesis that a greater modulation of performance and brain activation should be present in the treated side as a result of the interplay between expectancy and attention.

## Methods

### Participants

Sample size computation was performed with G-Power 3.1 (Faul et al. 2007), considering F tests for a within-between interaction with three groups and two sessions. Assuming an anticipated effect size *f* of 0.25 (considered medium by Cohen 1988), α error probability of 0.05, power (1 − β error probability) of 0.80, correlation among repeated measures of 0.5 and nonsphericity correction ε of 1, the resulting sample size is 42. To prevent a reduction of power due to potential dropouts, we recruited a total of 48 healthy subjects. Participants were recruited from the student population of the University of Verona (Italy) and randomly assigned to 3 groups of 16 subjects each: placebo group (mean age: 21.8 ± 2.2; 7 women; 14 right-handed), control transcutaneous electrical nerve stimulation (TENS) group (mean age: 21.8 ± 2.4; 7 women; 13 right-handed), control NoTENS group (mean age: 22.6 ± 4.0; 6 women; 15 right-handed).

Prior to the experiment, participants were screened for any contraindications to TMS and for neurological or psychiatric disorders (Rossi et al. 2009). Participants were not taking any medications at the time of the experiment and had normal or corrected to normal vision. Written informed consent was obtained from all participants prior to study participation. The study was approved by the Human Research Approval Committee (CARP 17.R1/2022) of the University of Verona and conducted in accordance with the Declaration of Helsinki.

### Simple reaction time motor task

Participants sat on a comfortable chair in front of a PC monitor (ASUS Full HD; screen size: 29 × 38.6 cm; screen resolution: 1280 × 1024 pixels) with the forearms relaxed on a table. They were asked to perform a simple reaction time (RT) motor task by abducting the thumb as quickly as possible after the appearance of a visual cue (i.e. a black circle, 2.5 cm diameter) presented in the center of the PC monitor on a gray background (Chen et al. 1998, Morgante et al. 2011, Cirillo et al. 2021). RT was defined as the latency between the presentation of the visual cue and the electromyographic (EMG) onset of movement recorded with surface electrodes from the abductor pollicis brevis (APB), the muscle directly involved in the task. The EMG onset was defined as the time point at which the EMG signal amplitude exceeded a predetermined threshold, indicating the beginning of muscle activation. In our study, the threshold was set at 2 SD above the baseline EMG amplitude, defined as the EMG recorded during the 50 ms preceding the onset of the visual cue.

**Figure 1.**
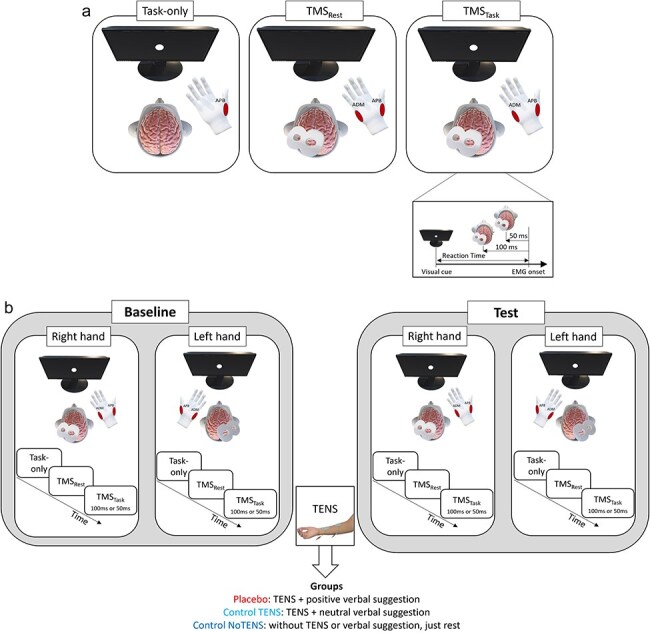
Schematic representation of the motor task and the procedure. (a) The simple RT motor task consisted of 10 rapid thumb abduction movements after the presentation of a visual go signal (a white dot appearing on the screen). In the Task-only condition, participants performed the motor task alone with the right or left hand with a blocked design and a counterbalanced order across participants. In the TMS_Rest_ condition, the TMS pulse was applied over the left or right M1 (depending on the hand performing the task) while the subjects were at rest. In the TMS_Task_ condition, the TMS pulse was delivered over the left or right M1 (depending on the hand performing the task) while the subjects performed the motor task. In this condition, the TMS pulse could be randomly delivered at 100 ms or 50 ms before the onset of the EMG. MEPs were recorded from the APB and ADM muscles of the right or the left hand. The APB was the muscle of interest, being involved in the task, while the ADM was a control muscle. (b) The three conditions (Task-only, TMS_Rest_, TMS_Task_) were performed before (Baseline) and after (Test) the placebo manipulation or the control procedures.

At the beginning of each experimental phase, participants were asked to perform 10 trials of the motor task (i.e. 10 thumb abduction movements). An interval of 8 s (± 1) was inserted between two consecutive trials (Cirillo et al. 2021). We called this condition “Task-only.” By averaging the RT of the 10 trials, we obtained the mean RT for each participant which was used not only to have a measure of performance but also to calculate the time window in which to apply the TMS pulse necessary to evaluate the premovement facilitation (details are reported in the procedure) (Fig. 1a). The motor task was performed separately with the right and the left hands in a counterbalanced order across participants.

### Transcranial magnetic stimulation

Single-pulse TMS was applied over the M1 contralateral to the hand performing the task (either the right or the left) with a blocked design and with a counterbalanced order across participants. The TMS pulses were delivered through a standard figure-of-eight coil with mean loop diameter of 90 mm, connected to a STM 9000 stimulator (EB Neuro). The coil was positioned tangentially to the skull at an angle of 45° to the sagittal plane.

Surface EMG was recorded from the APB and the abductor digiti minimi (ADM) muscles by means of unipolar self-adhesive Ag/AgCl electrodes (1.5 × 2.5 cm) in a belly-tendon montage. The APB was considered the muscle of interest, being directly involved in the task, while the ADM served as the control muscle (Duqué et al. 2010, Cirillo et al. 2021). The ground electrode was placed on the palm. EMG signals were amplified (×1000), filtered by bandpass from 10 to 500 Hz plus a notch filter set at 50 Hz (LabChart 8 pro, ADInstruments Co.), sampled at the frequency of 2 kHz, and digitized by PowerLab 16/35 (ADInstruments Co.). EMG signals were analyzed in real-time and offline with MATLAB 2014a (MathWorks Inc.).

The APB optimal scalp position was identified by moving the coil in small steps around the vertex and by delivering TMS pulses at 50% of the maximum stimulator power until stable and maximal MEP could be evoked in the APB muscle. The resting motor threshold (RMT) was defined for each subject and each hemisphere as the lowest stimulus intensity able to evoke an MEP with an amplitude of at least 50 μV in 5 out of 10 trials in the contralateral APB muscle (Temesi et al. 2014).

During the experiment, single-pulse TMS was delivered at 120% of the RMT in two conditions: at rest (TMS_Rest_) and during the motor task (TMS_Task_). In the TMS_Rest_ condition, 10 TMS pulses were applied over the right and left M1 while participants were at rest. This allowed us to measure the baseline corticospinal excitability of each participant. In the TMS_Task_ condition, TMS pulses were delivered during the motor task. This condition consisted of a total of 20 randomly presented trials: in 10 trials the TMS pulse was delivered 100 ms before the movement onset (TMS_Task100_) and in 10 trials the TMS pulse was delivered 50 ms before the movement onset (TMS_Task50_) (Fig. 1a). Since our protocol comprised several conditions (i.e. rest and active, right and left hand, baseline and test sessions), 10 TMS pulses per condition appear to provide an optimal balance between feasibility and reliable measurements (Cavaleri et al. 2017, Biabani et al. 2018, Cirillo et al. 2021).

TMS pulses were automatically triggered with MATLAB through a USB-6009 (National Instruments Corporation). As anticipated above, the time to apply the TMS pulse was derived from the mean RT obtained from the 10 trials of the simple RT motor task (Task-only) for each participant at the beginning of each session. Like in the Task-only condition, we considered an interval of 8 s (± 1) between two consecutive trials (Cirillo et al. 2021).

The neurophysiological parameter considered in this study was the peak-to-peak MEP amplitude (mV) recorded from the APB and ADM muscles. To rule out any effect of prestimulus EMG activity on MEP amplitude, prestimulation EMG activity was calculated on both muscles as the root mean square of the EMG over 50 ms prior to the TMS pulse.

### Procedure

After a short familiarization with the motor task (three trials), participants underwent the three conditions described above, presented always with the same blocked order: Task-only, TMS_Rest_, and TMS_Task_ (Fig. 1b). The blocked order was chosen based on previous studies on premovement facilitation (Chen et al. 1998, Morgante et al. 2011, Cirillo et al. 2021), in which the “Task-only” condition must inevitably always be performed at the beginning of the procedure to determine the individual’s mean RT used to apply the subsequent TMS pulse (at 100 ms or 50 ms before movement onset). For the two TMS conditions (i.e. TMS_Rest_ and TMS_Task_), we again used a blocked design because the TMS_Rest_ condition was conceived of as a “baseline” assessment of corticospinal excitability and counterbalancing the order of the TMS conditions would have carried out the potential risk of having varying levels of “baseline” corticospinal excitability across participants, as demonstrated by previous evidence where the application of consecutive TMS pulses over M1 induced cumulative changes in neural activity, resulting in increased corticospinal excitability (Pellicciari et al. 2016).

The conditions were separated by a time window of about 2 min and were performed in two sessions: baseline and test (Fig. 1b). The baseline session served to evaluate performance and premovement facilitation before any manipulation. Between the baseline and test session, the placebo group underwent a placebo manipulation, consisting of the application of 10 Hz transcutaneous electrical nerve stimulation (TENS) with two surface electrodes (1.5 cm diameter) over the volar surface of the right forearm. This stimulation *per se* does not produce any enhancing effects on motor performance (Fiorio et al. 2014). Nonetheless, the placebo group received positive verbal information about the effect of TENS on speeding-up thumb movements at the motor task. TENS intensity was adjusted so that participants felt a slight tingling sensation on the skin, though without any muscle contraction. The slight sensation served to convince participants that the device was active, thus boosting their expectation of TENS effectiveness. After 4 min, participants performed the test session. This time window appears to be adequate to let participants believe in the effectiveness of the device in the experimental condition (Fiorio et al. 2022, Emadi Andani et al. 2024). For consistency, the same time between sessions was maintained also in the control groups. During the test session, the TENS electrodes were still attached to the forearm, but with the stimulation intensity at zero level. This procedure served to maintain subjects’ belief about TENS effectiveness throughout the test session, without any confounding factor linked to skin sensation.

Two control groups (control TENS and control NoTENS), not exposed to any placebo manipulation, differed in terms of verbal information and procedures received. The control TENS group received TENS over the right forearm in the same (inert) modality of stimulation as the placebo group, though without any positive suggestion that TENS would enhance performance. In particular, participants of this group were told that they belonged to a control group, and therefore, the device was set at frequencies that have no effect on performance. This group served to control for potential effects due to the mere sight of TENS electrodes on task performance. Participants of the control NoTENS group received neither TENS nor any information about it and served to control for the natural course of performance throughout the procedure. Participants of this group had a rest of 4 min between the baseline and test sessions.

By applying TENS on the right forearm and asking participants to perform the task with the right and left hand, we had the secondary aim of investigating whether the placebo effect was specific for the treated side (i.e. right hand, left M1) or could generalize to the contralateral side.

### Subjective ratings

As a manipulation check for our placebo procedure, before the test session subjects were asked to judge how they expected their performance to change compared to baseline, by using a numerical rating scale (NRS), ranging from −3 (“much slower than before”) to +3 (“much faster than before”), with 0 denoting “like before.” Moreover, at the end of the experiment subjects were asked to rate the perceived effectiveness of TENS, on a 10 cm visual analog scale (VAS) ranging from 0 (“not effective at all”) to 10 (“very much effective”). Finally, after the motor task subjects were asked to evaluate their own performance on a VAS ranging from 0 (“very slow”) to 10 (“very fast”).

Importantly, the experimenter (M.B.), who was in direct contact with the participants, was responsible for giving them the information sheet, collecting signed informed consent, instructing participants on the RT task and collecting subjective scores. This experimenter was completely unaware of the group assignment, which was instead performed by a third researcher (M.E.A), who was never present in the laboratory, thus ensuring for the double-blind nature of our design. The experimenter who applied the TMS pulse (F.D.D.) was not blind, since he also applied the placebo or control intervention. For these reasons, we can exclude any experimenter bias on variables sensitive to it, such as RTs and subjective scores.

### Data handling

Trials of the TMS_Task_ conditions were inspected for muscle contraction preceding the TMS pulse, defined as EMG activity greater than 2 SD of the mean value recorded in the rest condition. Prestimulation EMG activity was below this threshold in all TMS_Task_ trials. Afterwards, MEP amplitude was inspected to exclude outliers (i.e. values 2.5 × SD above or below the mean value for each subject in each session and condition). Following this procedure, for the right hand we removed 0.52% of trials in the placebo group, 0.73% in the control TENS group, and 0.63% in the control NoTENS group; for the left hand we removed 0.63% of trials in the placebo group, 0.94% in the control TENS group, and 0.31% in the control NoTENS group.

### Statistical analysis

The three groups were compared for gender distribution and hand-dominance by means of chi-squared test and for age and RMT with one-way analysis of variance. Behavioral and neurophysiological data were normally distributed (Shapiro–Wilk test, *P* > .061), with equal variances between groups (Levene’s test, *P* > .085). RTs obtained in the Task-only condition were analyzed by means of repeated measures analysis of variance (rmANOVA) with Phase (baseline and test) as within-subjects factor and Group (placebo, control TENS, and control NoTENS) as between-subjects factor. This analysis allowed to determine whether the placebo procedure was effective in modifying participants’ performance without any interference induced by the TMS pulse.

MEP amplitude and prestimulation EMG activity were analyzed, separately for the APB and ADM muscles, by means of rmANOVAs, with Phase (baseline and test) and Time (TMS_Rest_, TMS_Task100_, TMS_Task50_) as within-subjects factors and group as between-subjects factor.

Subjective variables were analyzed with nonparametric tests due to the ordinal nature of the data. In particular, the Wilcoxon signed rank test was used to compare the sessions within each group separately, and the Kruskal–Wallis test to compare the groups within each session separately.

All analyses were conducted for the right and left hand, separately. Post-hoc comparisons were performed with paired- and independent-samples *t*-tests for parametric analyses and Mann–Whitney test for nonparametric analyses. The effect size of all significant results was reported with partial eta-square (η_p_^2^) and Cohen’s d for rmANOVA and parametric post-hoc tests, respectively (Fritz et al. 2012) and with Kendall’s W, and r for Kruskal–Wallis, and nonparametric post-hoc tests, respectively (Tomczak and Tomczak 2014).

Bayes factors quantifying the evidence in favor of the alternative hypothesis (BF_10_) were computed (van Doorn et al. 2020). A Bayes factor BF_10_ > 3 was interpreted as substantial, and a BF_10_ > 10 as strong evidence against the null hypothesis.

Spearman’s coefficient of correlation was used to correlate the subjective data with the change (Δ) in neurophysiological and behavioral data from the baseline to the test sessions computed for each participant as follows:


$${\Delta {\rm {RT = R}}}{{\mathrm{T}}_{{\mathrm{Baseline}}}}{\mathrm{- R}}{{\mathrm{T}}_{{\mathrm{Test}}}}$$

$${\Delta {\rm {MEP = ME}}}{{\mathrm{P}}_{{\mathrm{Baseline}}}}{\mathrm{- ME}}{{\mathrm{P}}_{{\mathrm{Test}}}}$$

where ΔRT or ΔMEP is the difference between the baseline and test phase in RTs (obtained in the Task-only condition) or MEPs (measured at 100 ms and 50 ms before EMG onset). The delta is informative of the amount of change in performance and corticospinal excitability before and after the procedure and can be considered as a proxy of the placebo effect. Additionally, we plotted ΔRT and ΔMEP together and analyzed their relationship with a quadrant analysis described in the Supplementary material. The ΔMEP was also submitted to a further analysis by including Muscle and Hand as factors (see Supplementary material for details and results).

Bonferroni correction for multiple comparisons was applied where necessary and the significance level was set at *P* < .050.

## Results

The three groups did not statistically differ for gender, hand-dominance (*P* > .669, for both), age, and RMT (right hemisphere, placebo: 55.37 ± 7.89, control TENS: 61.09 ± 10.54, control NoTENS: 59.74 ± 6.32; left hemisphere, placebo: 56.98 ± 9.32, control TENS: 58.8 ± 8.62, control NoTENS: 58.33 ± 10.75; *P* > .163, for both hemispheres).

### Reaction times

**Figure 2.**
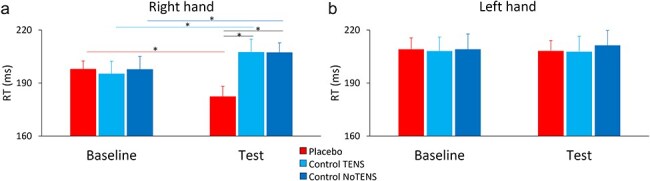
Mean RTs in the Task-only condition for the right and left hand. (a) RTs on the right hand were shorter in the test compared to the baseline session in the placebo group (red bar), while they were longer in the test compared to the baseline in the control TENS (light blue bar) and control NoTENS groups (dark blue bar). In the test session, the RTs of the placebo group were shorter compared to the two control groups (black lines). (b) RTs on the left hand were stable across sessions and groups. Asterisks represent *P* < .05. Error bars represent the standard error of the mean.

On the right hand, the significant interaction Phase × Group (F_(2,45)_ = 13.935, *P* < .001, η_P_^2^ = 0.382) revealed that RTs were shorter in the placebo group compared to the two control groups, specifically in the test session (t_(30)_ > 2.56, *P* < .029, Cohen’s d > 0.45, BF_10_ > 3.40). Furthermore, in the placebo group RTs were shorter at test than at baseline (t_(15)_ = 3.31, *P* < .001, d = 0.59, BF_10_ = 9.52), while in the two control groups RTs were longer at test compared to baseline (t_(15)_ > 2.86, *P* < .036, d > 0.51, BF_10_ > 4.31) (Fig. 2a). These findings suggest that the procedure was effective in speeding up RTs in the placebo group. The effect of Phase or Group was not statistically significant (*P* > .279). No statistically significant result was found on the left hand (*P* > .925, BF_10_ < 0.26), indicating that the improvement of RTs in the placebo group was specifically localized on the hand on which the placebo device was applied (Fig. 2b). Additional analyses are reported in the Supplementary material and Supplementary  Fig. 1.

### Neurophysiological data

**Figure 3.**
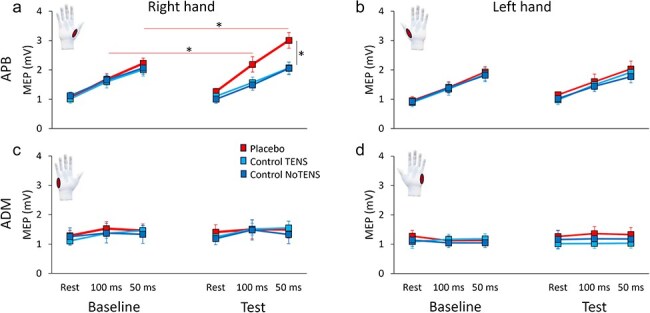
Mean amplitude of the MEP across sessions and conditions. (a) The amplitude of the MEPs recorded from the right APB was greater in the test session compared to the baseline in the placebo group (solid red lines) both at 100 ms and 50 ms before EMG onset. In the test session, the MEP amplitude of the placebo group was higher than that of both control groups at 50 ms before EMG onset (vertical black line). (b) Left APB MEP amplitude did not change across sessions, groups, and conditions. (c) Right ADM MEP amplitude was stable across sessions, groups, and conditions. (d) Left ADM MEP amplitude did not change across sessions, groups, and conditions. Asterisks represent *P* < .05. Error bars represent the standard error of the mean.

**Figure 4.**
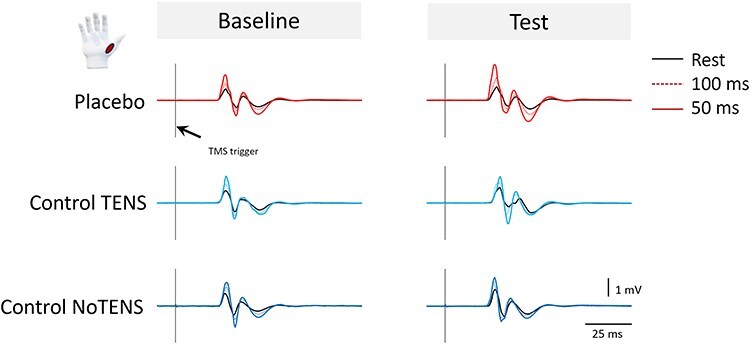
Representative traces of the motor evoked potentials. (a) Neurophysiological traces recorded in the baseline session from the APB muscle of the right hand in one subject of the placebo group (red traces), one subject of the control TENS group (turquoise traces), and one subject of the control NoTENS group (blue traces). (b) Neurophysiological traces recorded in the test session. Vertical lines indicate TMS pulse delivery. Black lines represent MEPs at rest, colored dashed lines represent MEPs for TMS pulse delivered 100 ms before EMG onset and colored continuous lines the MEPs for TMS pulse delivered 50 ms before EMG onset. MEP amplitude is reported in mV and trace duration is shown in ms. As illustrated in the figure, MEP amplitude was higher 50 ms and 100 ms before EMG onset, indicative of premovement facilitation. In the test session, the subject of the placebo group presents an amplification of the premovement facilitation before EMG onset.

On the right hand, APB MEP amplitude was greater in the TMS_Task100_ than in the TMS_Rest_ condition, and it was also greater in the TMS_Task50_ compared to the other two conditions (main effect of Time: F_(2,90)_ = 67.481, *P* < .001, η_P_^2^ = 0.600; post-hoc comparisons: for all, *P* < .001). This finding certifies the presence of premovement facilitation at 100 ms and 50 ms before EMG onset and is in line with previous studies (Cirillo et al. 2021). More interestingly, the interactions Phase × Group (F_(2,45)_ = 11.936, *P* < .001, η_P_^2^ = 0.347) and Phase × Time × group were significant (F_(4,90)_ = 2.668, *P* = .037, η_P_^2^ = 0.106). Since the latter interaction encompassed the former, it was further analyzed. Consistent with our hypotheses, post-hoc comparisons revealed that APB MEP amplitude was greater in the placebo group compared to both control groups in the test session of the TMS_Task50_ condition (t_(30)_ > 2.70, *P* < .033, d > 0.95, BF_10_ > 4.46). Furthermore, in the placebo group a greater APB MEP amplitude was found at test compared to baseline at TMS_Task50_ and TMS_Task100_ (t_(15)_ > 3.13, *P* < .021, d > 1.11, BF_10_ > 6.90) (Fig. 3a). On the left hand, MEP amplitude increased across times (main effect of Time: F_(2,90)_ = 74.25, *P* < .001, η_P_^2^ = 0.623), certifying the presence of the premovement facilitation (Cirillo et al. 2021), but no significant effect was found for Phase, Group, or their interactions (*P* > .062, BF_10_ < 0.84), indicating that the placebo effect did not affect the left hand (Fig. 3b). No significant effect was found on the ADM (for all factors, *P* > .298, BF_10_ < 0.43) (Fig. 3c and d). Representative MEPs from the APB are shown in Fig. 4.

Analysis of the prestimulation EMG activity disclosed no significant effect (for all factors, *P* > .282), indicating that MEP amplitude was not influenced by differences in preceding EMG activity.

### Subjective data and correlation

**Figure 5.**
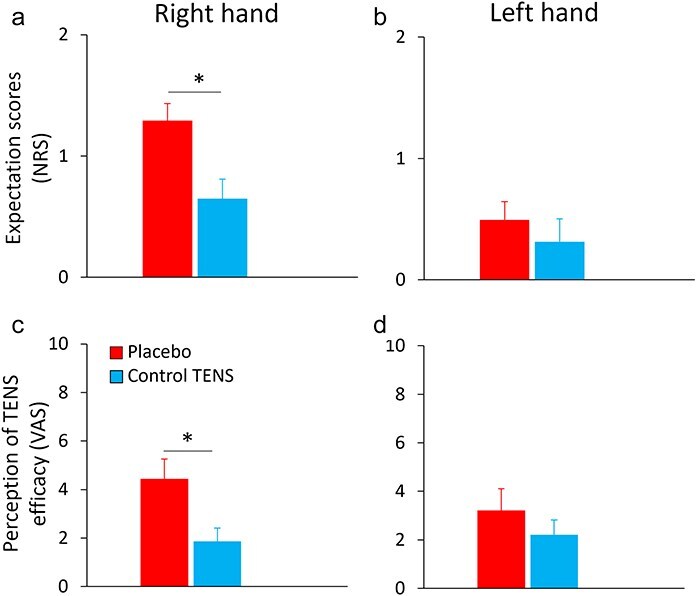
Mean values of the subjective variables. (a) Expectation scores on the right hand were higher in the placebo group (red bar) compared to the control TENS group (light blue bar). (b) No difference was found between groups on the left hand. (c) Perception of TENS effectiveness on the right hand was higher in the placebo group compared to the control TENS group (black line). (d) No difference was found between groups on the left hand. Asterisks represent *P* < .05. Error bars represent the standard error of the mean.

On the right hand, the placebo group reported higher expectation ratings compared to the control TENS group (Z = −2.26, *P* = .029, r = 0.40, BF_10_ = 6.67), while no difference was found on the left hand (*P* = .616, BF_10_ = 0.32) (Fig. 5a and b). Similarly, on the right hand, the placebo group reported higher ratings of TENS effectiveness compared to the control TENS group (Z = −2.43, *P* = .015, r = 0.43, BF_10_ = 3.29), with no difference on the left hand (*P* = .341, BF_10_ = 0.36) (Fig. 5c and d). These findings indicate that the placebo procedure was effective in inducing positive expectations and belief in TENS effectiveness in the placebo group.

Participants of the placebo group reported higher performance scores in the test than in the baseline session, only on the left hand (Z = −2.84, *P* = .004, r = 0.50, BF_10_ = 27.78).

A positive correlation was found in the placebo group between expectation scores and ΔRT on the right hand (rho = 0.542, *P* = .030, Supplementary Fig. S2). This correlation indicates that the higher the expectation of improvement, the greater the improvement at the motor task. Further analyses on the relation between ΔRT and ΔMEP are reported in the Supplementary materials and in Supplementary Figs. S3 and S4.

## Discussion

Taking advantage of the premovement facilitation paradigm, we demonstrate for the first time that the placebo effect can boost the excitability of the corticospinal tract before movement onset. Our results help refine the knowledge of the neurophysiological basis of the placebo effect in the motor domain and can be interpreted in the light of the active inference account.

### Premovement facilitation reveals a highly specific placebo effect before movement initiation

First of all, our results provide evidence that the paradigm was suitable for obtaining a reliable and clear premovement facilitation in all groups, as revealed by the increase in MEP amplitude in the muscle directly involved in the task (i.e. right and left APB) before movement initiation (i.e. 100 ms and 50 ms before EMG onset). The novel finding of the present study is that premovement facilitation turned out to be amplified in a very specific way after the placebo manipulation, thus suggesting a role of the placebo effect on the activation of the corticospinal tract from M1 prior to movement onset.

Previous studies using central (TMS and electroencephalography) or peripheral (EMG) techniques converged to indicate the existence of a top-down mechanism through which positive expectancy induced by placebo procedures could enhance the activity of the corticospinal system (Fiorio et al. 2014, Emadi Andani et al. 2015, Piedimonte et al. 2015, Fiorio 2018, Rossettini et al. 2018, Corsi et al. 2019). Taking advantage of the good spatial and temporal resolution of TMS (Garcia-Sanz et al. 2022), we were able to demonstrate that the placebo effect induced a precise amplification of premovement facilitation that was specific to the muscle involved in the task (i.e. APB) and localized on the treated side of the body (i.e. on the right). Indeed, the homologous muscle on the untreated side of the body (i.e. left APB) or adjacent muscles not involved in the task (i.e. right and left ADM) did not show any change in MEP amplitude.

This neurophysiological finding is consistent with the behavioral data, as the performance improvement was found specifically on the side of the body on which the placebo treatment was applied. The subjective data confirmed that our manipulation was effective in inducing positive expectations and beliefs about the effectiveness of TENS, particularly on the right hand, giving further support to the specificity of the placebo effect on the treated body part. The spatial localization of the placebo effect has also been observed in other domains, such as pain. For instance, a placebo analgesic effect was observed only on the participant’s arm treated with a placebo cream when expectancy was directed toward the same body part (Benedetti et al. 1999). Similarly, Colloca et al. (2008) confirmed a placebo analgesic effect localized to the treated hand that was associated with a specific neurophysiological reduction of the N2-P2 component, a brain wave evoked by laser stimulation and representing the emotional component of pain (Colloca et al. 2008). Altogether, this evidence suggests that when the placebo intervention is spatially localized, the resulting sensory, motor, or neurophysiological effects are specific to the body part or muscle treated.

It should be noted that in our study the placebo treatment had some characteristics that could have favored a “spatially localized effect.” First, the placebo consisted of a tangible device (i.e. TENS) that was clearly applied over a specific body part (i.e. the forearm). Second, the placebo device was expected to affect a task involving a restricted body district (i.e. the right thumb). Consequently, given these characteristics, it was reasonable to expect that the placebo effect could be explicitly localized to the right hand. Although predictable, this result represents the first experimental behavioral and neurophysiological evidence for a spatial localization effect of a placebo device in the motor domain. Therefore, our study could provide a benchmark for comparing the effects of less tangible types of placebos (e.g. oral pills, oxygen inhalation, etc.) on movements performed with one or multiple body districts. To date, only one study on pain (Kong et al. 2013) has tried to compare different types of placebos, demonstrating that verbally suggested placebo pills or sham needles can induce different responses and supporting the idea that the type of intervention could likely play a role in shaping the placebo effect.

### A dissociation between behavioral and subjective variables

While the placebo group showed a shortening of RTs on the right hand, the two control groups showed an increase in RTs from the baseline to the test session. A possible explanation for this result could be related to the mindset instilled in all participants from the beginning of the procedure. In fact, before signing the consent form, all participants read an information sheet in which the general objective of the project was passed off as research on a new TENS intervention to improve motor performance. This could have fueled certain expectations about the nature of the study. Therefore, while participants assigned to the placebo group found these expectations met, those assigned to the control groups may have had the feeling that the intervention had been denied (either because it had been applied at inert frequencies, as the control TENS group was told, or because they had not received TENS at all, as was the case with the control NoTENS group). We could reason that a sort of “negative mindset” was induced in the control groups, thus consequently worsening motor performance on the right hand in test session. This hypothesis should be tested in future studies in which a nocebo group will be included (i.e. a group that receives the same TENS device with negative verbal information). The lack of modulation on the left hand could be interpreted in the light of previous evidence showing that motor performance of the nondominant hand is typically slower than the dominant hand (Todor and Kyprie 1980, Todor et al. 1982, Heuer 2007). Since our participants were mostly right-handed (87.5% in the placebo group, 81.3% in the control TENS group, and 93.8% in the control NoTENS group), we can speculate that the left hand performance was overall slow, and this might have limited further worsening of RTs in the test phase, as a sort of “floor” effect. Future research with longer task durations may allow us to investigate whether performance impairment would occur in the control groups on the left hand.

Although they were objectively faster with their right hand, participants of the placebo group perceived themselves as faster with their left hand after the placebo procedure, hinting at a dissociation between the behavioral and subjective levels. Again, given that most participants were right-handed, we could hypothesize that they may have been imprecise in judging the performance of the left, nondominant hand, thus subjectively reporting an improvement that, however, was not objectively detected. A similar dissociation was described in a previous study in which participants reported perceiving the positive effects of a placebo treatment on performance despite the lack of effect detected by the objective measure of actual performance (Schwarz et al. 2015).

### The placebo effect through the lens of active inference

Our findings can be interpreted according to the active inference account (Adams et al. 2013). If we consider verbal suggestion as a contextual psychosocial factor that can shape internal predictions (Kaptchuk et al. 2020, Pagnini et al. 2023), we can hypothesize that the information provided to participants assigned to the placebo group could have acted as a reliable contextual cue regarding the achievable speed, thus modeling precise internal predictions about the consequence of the motor act (i.e. faster performance at the thumb abduction task). Therefore, individuals in the placebo group likely updated their internal predictions on the upcoming action based on the verbal suggestion received. In other words, the expectancy of being faster at the motor task could have induced a peculiar mindset characterized by positive and precise internal predictions about motor performance. The subjective data support this hypothesis, as the placebo group had a strong expectancy about the effects of TENS in speeding up RTs and a strong belief that TENS was an effective intervention.

This mindset could have impacted both the behavioral level, with faster movements in the placebo group, and the neurophysiological, with an amplified premovement facilitation. The latter could be interpreted as a sign of precise internal predictions about the upcoming movement outcome (i.e. faster RTs). In keeping with this interpretation, a previous TMS study showed that the presence of reliable prior information about an upcoming motor response (like, for instance, a visual cue indicative of the effector to be moved) induced an increase in MEP amplitude, specific for the muscle involved in the task (Mars et al. 2007). Consistently, another TMS study showed that MEP amplitude increased in the preparatory phase in trials characterized by valid cues compared to trials characterized by invalid cues (Bestmann et al. 2008).

### Limitations and concluding remarks

It should be acknowledged that our study presents some limitations. First, our sample was predominantly represented by right-handers and therefore we cannot clarify whether the side of the body on which the placebo device was applied interacted with handedness in determining the placebo effects observed here.

Second, we did not directly measure fatigue and fatigability in our study and therefore we cannot completely exclude the presence of fatigue in our participants, especially in the two control groups (Langner et al. 2010, Jaydari Fard et al. 2019). Nonetheless, we consider a potential effect of fatigue on our data very unlikely, because (i) the conditions in which participants were required to perform the RT task (i.e. the Task-only and the TMS_Task_) were interleaved by a rest period (i.e. TMS_Rest_ condition); (ii) the baseline and test sessions were interleaved by 4 min rest, thus further allowing for recovery; (iii) the abduction movements of the RT task were very brief and separated by an intertrial interval of about 8 s; (iv) the task did not require exerting maximum voluntary muscle contraction or certain levels of force, as typically required in fatigue inducing tasks (Enoka and Duchateau 2007). Considering all these aspects, we would exclude the effect of fatigue on our results.

Third, we focused on the corticospinal tract, leaving open the question on the role of other brain regions in modulating these effects. Namely, a reasonable hypothesis is that the neural bases of our findings may be related to a complex brain network involved in motor control. As anticipated above, motor-related brain areas have been already shown to play a role in the placebo effect (Fiorio 2018). In this regard, we hypothesize that the dorsolateral prefrontal cortex (DLPFC) may play a fundamental role, being a brain region involved in processing expectation and often being active in neuroimaging studies on the placebo effect (Wager et al. 2004, Kong et al. 2006, Watson et al. 2009, Krummenacher et al. 2010, 2010, Lui et al. 2010, Geuter et al. 2013, Jubb and Bensing 2013, Peciña et al. 2013, Egorova et al. 2015, Villa‐Sánchez et al. 2018). Interestingly, the DLPFC has functional connections with M1 and is involved, together with other frontal regions, in the cognitive control of motor behavior (Miller and Cohen 2001, Hasan et al. 2013). Therefore, our hypothesis is that the positive expectations induced by the placebo procedure might recruit the DLPFC and strengthen its projections to M1, thus enhancing corticospinal excitability before movement onset and consequently speeding up movement execution.

These limitations notwithstanding, our study represents the first demonstration that the corticospinal tract from M1 can be boosted by the placebo effect before movement onset, thus, in turn, facilitating movement preparation and execution. This study contributes to broadening our understanding of how expectancy influences motor behavior and the complex neural circuits underlying motor control and provides experimental support for the active inference account. Albeit cautiously, this investigation encourages future advances in the clinical field, where tailored placebo procedures could be used to address individuals’ spared motor resources, potentially supporting motor recovery.

## Supplementary Material

nsaf014_Supp

## Data Availability

The data underlying this article will be shared upon reasonable request to the corresponding author.
