# Supplementary material for: Premovement activity in the corticospinal tract is amplified by the placebo effect: an active inference account
Source: Soc Cogn Affect Neurosci. 2025 Feb 1;20(1):nsaf014. doi: 10.1093/scan/nsaf014 (PMC11799862; doi:10.1093/scan/nsaf014)
Supplement: nsaf014_Supp [file nsaf014_supp.zip › scan-24-190-File007.docx]

**Supplementary material**

**Premovement activity in the corticospinal tract is amplified by the placebo effect: an active inference account**

Mehran Emadi Andani^1*^, Miriam Braga^1^, Francesco Da Dalt^1^, Alessandro Piedimonte^2^, Elisa Carlino^2^, Mirta Fiorio^1*^

^1^ Department of Neurosciences, Biomedicine and Movement Sciences, University of Verona, Verona, Italy

^2^ Department of Neuroscience, University of Torino, Torino, Italy

**Reaction times across conditions**

In order to check whether RTs changed when the motor task was executed alone (Task-only) with respect to the condition in which TMS was delivered (TMS_Task_), we ran an additional rmANOVA on RTs with Phase (baseline, test), and Condition (Task only, TMS_Task100_, TMS_Task50_) as within-subjects factors and group (placebo, control TENS, control NoTENS) as between-subjects factor.

On the right hand, a main effect of Time was found (F(2,90) = 101.67, p < 0.001, η_P_^2^ = 0.692), due to longer RTs in the TMS_Task50_ compared to TMS_Task100_ condition, in which RTs were longer compared to the Task-only condition (for all, p < 0.013, d > 0.75). The interaction Phase × Condition was also significant (F(2,90) = 5.157, p = 0.008, η_P_^2^ = 0.103). Post-hoc comparisons showed that RTs were longer in the TMS_Task50_ compared to the TMS_Task100_ condition both in the baseline (for all, p < 0.016, d > 0.73) and test sessions (for all, p < 0.019, d > 0.71). Moreover, RTs were longer in the TMS_Task100_ and TMS_Task50_ compared to the Task-only condition (for all, p < 0.037, d > 0.61). The interaction Phase × Group was also significant (F(2,45) = 9.48, p < 0.001, η_P_^2^ = 0.296), due to shorter RTs at test than at baseline only in the placebo group (p = 0.001, d = 0.94) (Fig. S1A). The effect of Condition was found significant on the left hand (F(2,90) = 57.66, p < 0.001, η_P_^2^ = 0.562), due to longer RTs across conditions (for all, p < 0.013, d > 0.89). No other statistically significant effect was found on the left hand (p > 0.075) (Fig. S1B).

These results indicate that the placebo procedure was successful in speeding up RTs, even when the TMS pulse was delivered, suggesting a robust improvement in performance.


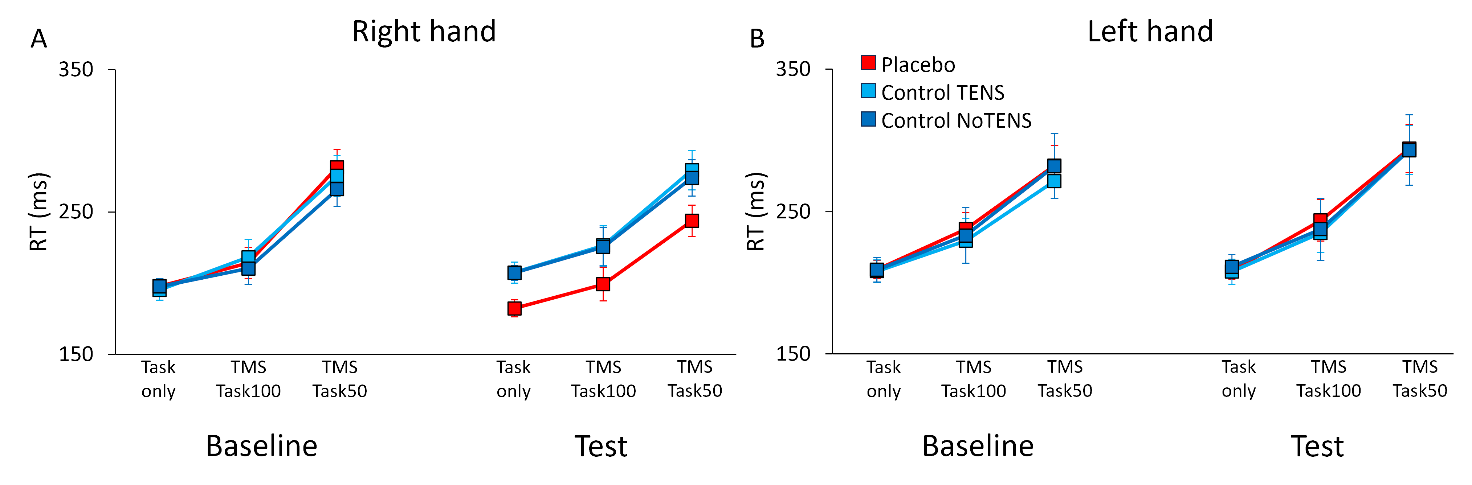


**Figure S1. Mean reaction times in different conditions**. **A)** On the right hand, RTs were longer in the TMS_Task50_ than in the TMS_Task100_ condition, which in turn were longer than in the Task-only condition. This pattern was present both at baseline and in the test session. In the placebo group, the RTs in the test session were shorter than in the baseline, in all conditions. **B)** On the left hand, no difference was found in RTs across sessions, conditions, and groups. Error bars represent the standard error of the mean.


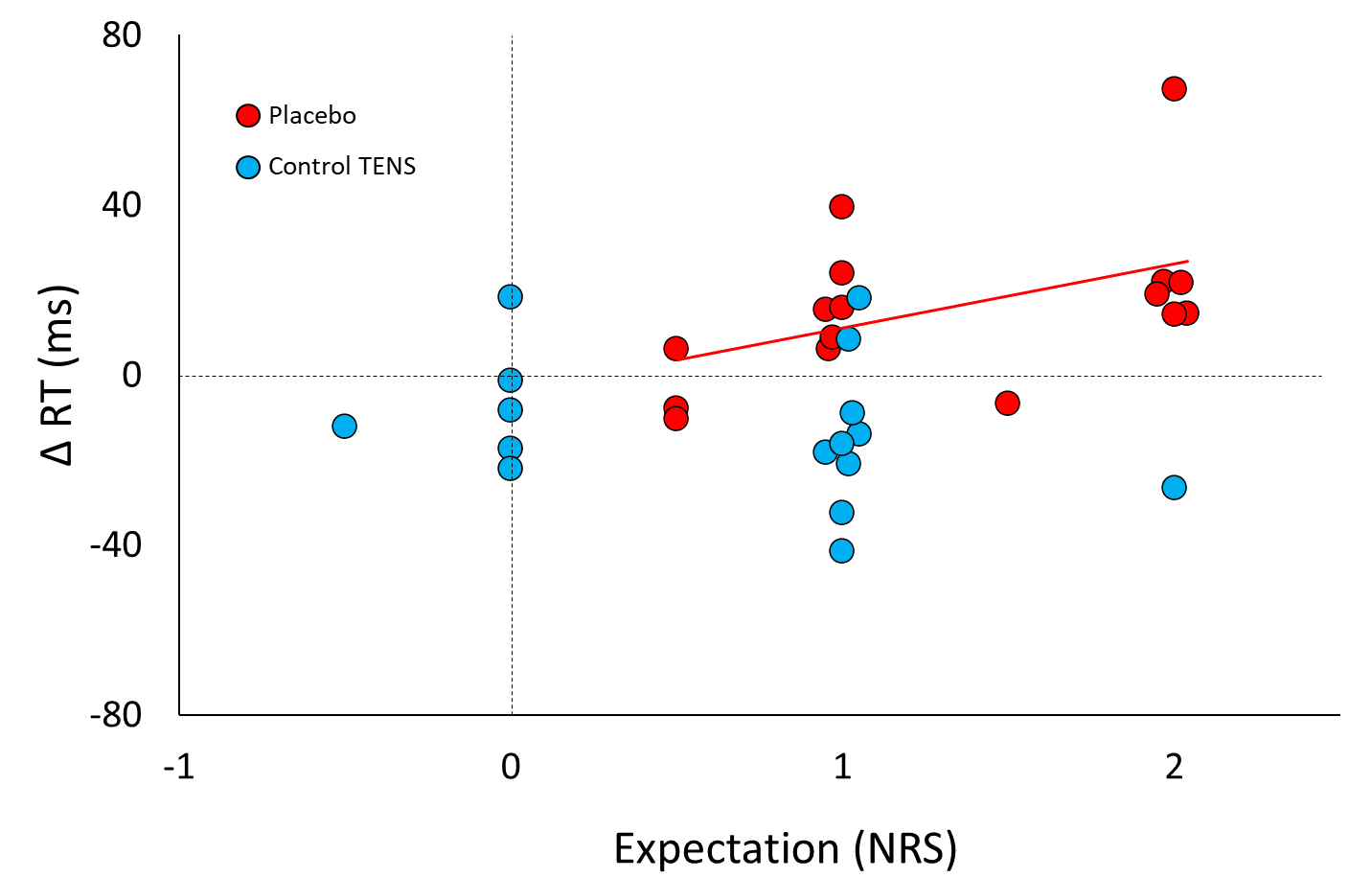


**Figure S2. Correlation between expectation scores and change in RTs.** A significant positive correlation was found between ΔRT (baseline – test) and expectation scores in the placebo group (red dots), whereas no correlation was found in the control TENS group (light blue dots). In this figure, some data points have been artificially shifted to the left or right to improve the representation.


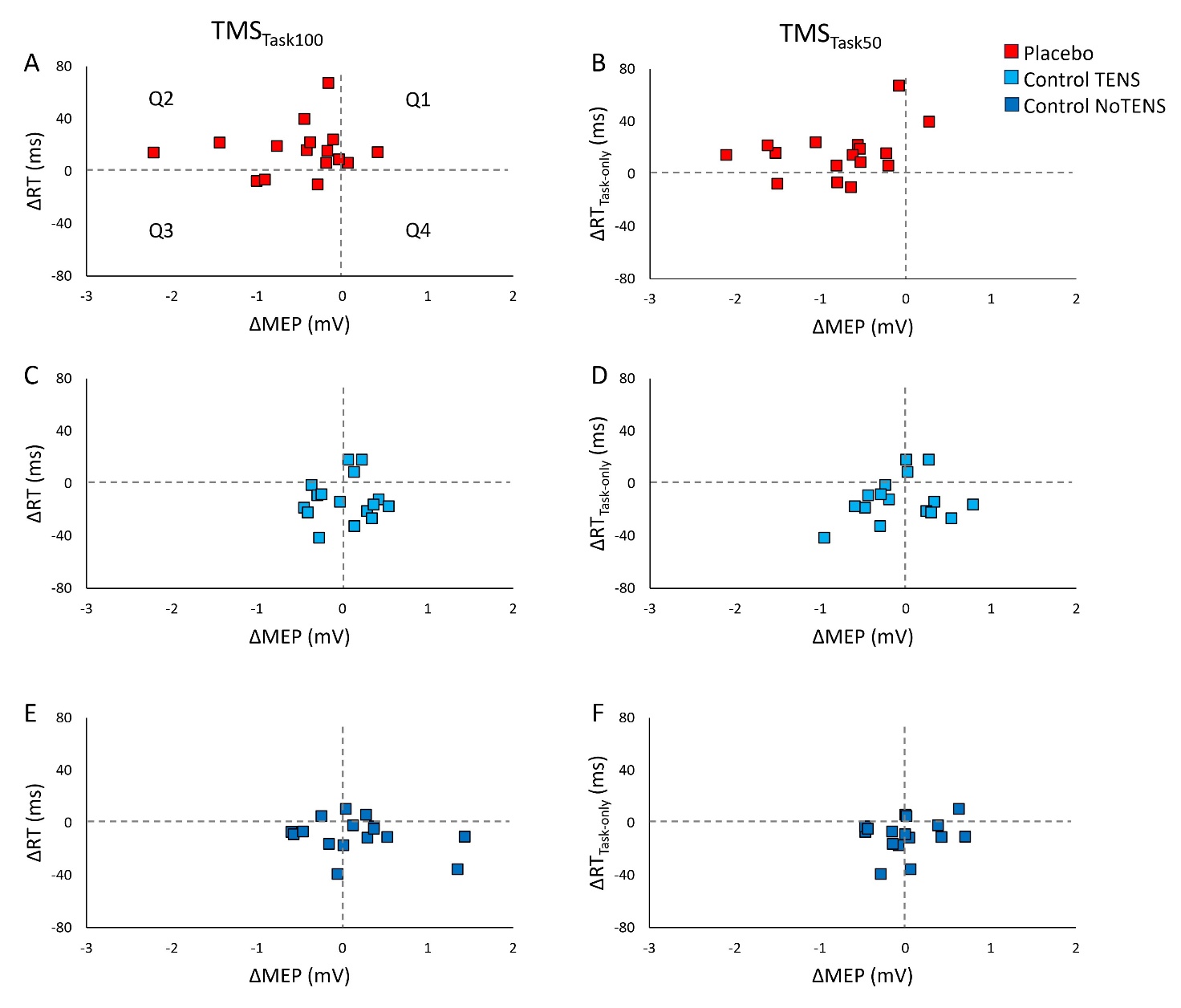


Q1

Q2

Q4

Q3

**Figure S3. Relationship between changes in RTs and MEPs across groups.** ΔRT (baseline – test) obtained in the Task-only condition is plotted against ΔMEP (baseline – test), measured form the APB muscle at 100 ms (left panels) and 50 ms (right panels) before EMG onset, in the placebo (red squares, A, B), control TENS (light blue squares, C, D), and control NoTENS (blue squares, E, F) groups. The data points appear to be distributed in different quadrants across groups. The quadrants are numbered counterclockwise from the top right (Q1) to the bottom-right (Q4). Q1 and Q4 indicate positive ΔMEP, while Q2 and Q3 indicate negative ΔMEP. Additionally, Q1 and Q2 represent positive ΔRT, whereas Q3 and Q4 represent negative ΔRT. The placebo group exhibited a distinct shift of data points toward Q2 (top-left quadrant), suggesting a trend in which positive ΔRTs align with negative ΔMEPs. This distribution may reflect a unique response profile specific to the placebo group, a trend that is not present in either of the control groups. Q = quadrant.

**Quadrant analysis**

We conducted a quadrant analysis to determine whether the three groups (placebo, control TENS, and control NoTENS) were systematically associated with a specific distribution of data points in one of the four quadrants of Figure S3. To this aim, the frequencies of the data points in each quadrant were compared between groups by means of Chi-square test. A significant Chi-square test indicates that the distribution of data across quadrants is not random but influenced by the group to which they belong.

Results revealed significant differences in the frequency of data between the placebo and both control groups in Q2 (TMS_Task100_: χ²(1, N = 32) > 13.333, p < .001; TMS_Task50_: χ²(1, N = 32) > 19.2, p < .001) and Q4 (TMS_Task100_: χ²(1, N = 32) > 7.385, p < 0.021; TMS_Task50_: χ²(1, N = 32) > 5.926, p < 0.045). No significant differences were observed in Q1 and Q3, nor between the two control groups across all quadrants (p > 0.189). These results indicate that quadrant occupancy varied significantly across groups. Namely, in the placebo group, over 68.75% of data points fell within Q2, indicating a pattern of higher MEP amplitude and shorter RT in the test phase compared to baseline. In contrast, the control groups exhibited a more dispersed distribution of data across quadrants, with fewer data in Q2 (< 6.25%). Additionally, over 31.25% of data in both control groups fell within Q4, while no data from the placebo group was observed in Q4. Data in Q4 indicates lower MEP amplitude and longer RT in the test phase compared to baseline. The normalized frequency of data points, calculated as a percentage of the total data points for each group, is illustrated in Figure S4.


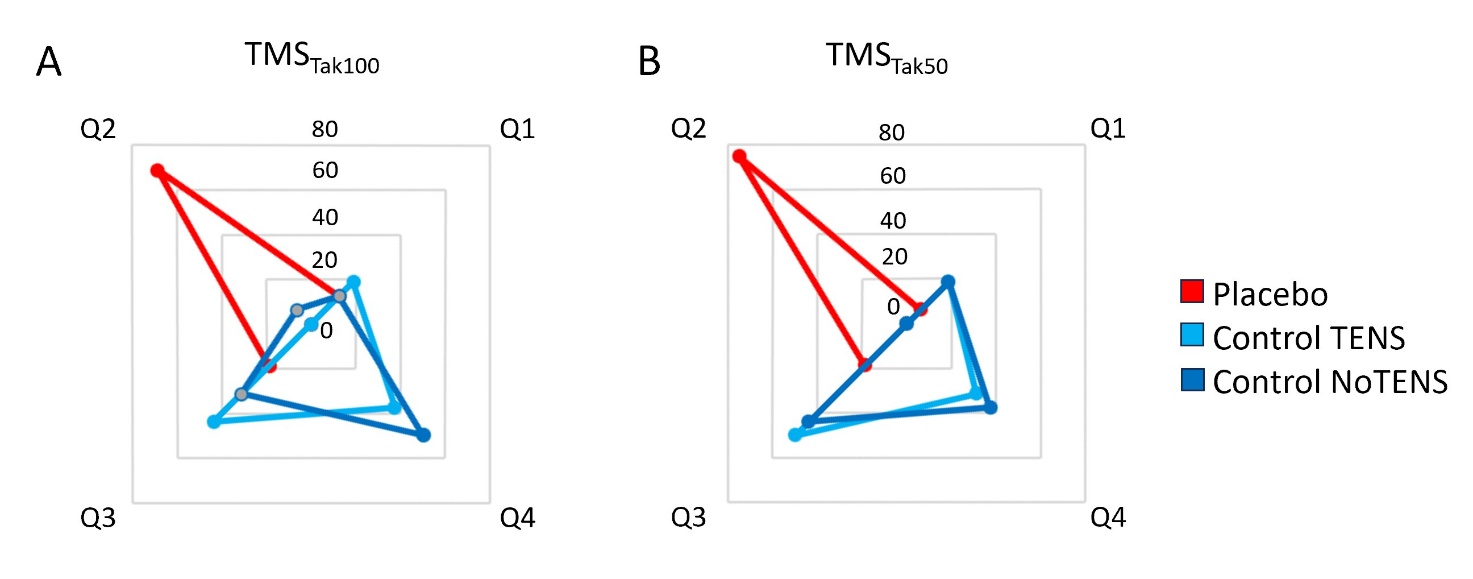


**Figure S4. Radar chart on the frequency percentage of data points across quadrants.** Panels A and B present results for TMS_Task100_ and TMS_Task50_, respectively. The number of data points in each quadrant is normalized to the total number of data points for each group (N = 16). A distinct pattern emerges between the placebo and the control groups, with no significant differences between the two control groups. In the placebo group, over 68.75% of data points are concentrated in Q2, whereas Q2 contains less than 6.25% of data points in the control groups.

**Analysis of change in MEP amplitude**

A repeated measures ANOVA was conducted on the change of MEP amplitude computed as difference between the baseline and test sessions (ΔMEP: baseline – test). This analysis included Muscle (APB, ADM), Hand (Left, Right) and Time (TMS_Rest_, TMS_Task50_, TMS_Task100_) as within-subjects factors and Group (placebo, control TENS, control NoTENS) as between-subjects factors. The analysis revealed a significant interaction Muscle × Hand × Group (F(2, 45) = 5.786, p = 0.006, ηp² = 0.205). Post-hoc comparisons showed that the ΔMEP was significantly higher in the placebo group compared to both control groups for the APB muscle of the right hand (p < 0.006, d > 0.81 for both comparisons). Additionally, within the placebo group, ΔMEP was significantly greater on the right APB than the left APB (p = 0.004, d = 0.76). Finally, in the placebo group, ΔMEP was significantly higher on the right APB than the right ADM (p < 0.001, d = 0.97). No significant effects were observed for the remaining factors or interactions (p > 0.069). This additional analysis confirms the main results that the placebo procedure is specifically effective on the right hand and on the muscle involved in the task.
